# *Q* allele overexpression and alternative splicing can improve wheat yield by increasing thousand-kernel weight and grain number per spike

**DOI:** 10.1186/s12870-026-08188-4

**Published:** 2026-01-24

**Authors:** Qingcheng Li, Yazhen Fan, Yang Li, Lu Lei, Shengling Zhang, Simin Xie, Lifen Lu, Xin Hu, Fengqi Lv, Zhenru Guo, Xin Chen, Linlin Zhou, Yan Wang, Jing Zhu, Li Kong, Xin Yuan, Jie Deng, Binjie Xu, Xinyou Cao, Yunfeng Jiang, Guoyue Chen, Qiang Xu, Qiantao Jiang, Yuming Wei, Youliang Zheng, Qing Chen, Pengfei Qi

**Affiliations:** 1 State Key Laboratory of Crop Gene Exploration and Utilization in Southwest China, Sichuan Agricultural University, Chengdu, 611130 China; 2 Triticeae Research Institute, Sichuan Agricultural University, Chengdu, 611130 China; 3 Innovative Institute of Chinese Medicine and Pharmacy, Chengdu University of Traditional Chinese Medicine, Chengdu, 611137 China; 4 Crop Research Institute, Shandong Academy of Agricultural Sciences, Jinan, 250100 China

**Keywords:** Alternative splicing, *Q* gene, Yield, Quality, Wheat breeding

## Abstract

**Background:**

Thousand-kernel weight and grain number per spike are key determinants of grain yield. *Q* is one of the most important domestication genes in wheat. A missense mutation (C3129T) at the microRNA172-binding site of *Q* (*Q*^c1^ allele) significantly increases the thousand-kernel weight, but also results in extremely compact spikes, leading to decreases in grain number per spike and yield, which is unfavorable for breeding and wheat production.

**Results:**

We characterized a new *Q* allele (*Q*^*c1*^*-Q1*) obtained by treating the *Q* gene mutant line *S-Cp1-1* (carrying *Q*^*c1*^) with ethyl methanesulfonate. A comparison with the domesticated *Q* allele widely distributed in modern common wheat cultivars revealed that *Q*^*c1*^*-Q1* contains two missense mutations: C3129T (as in *Q*^*c1*^) and G542A in the splice site of the second intron. G542A results in alternative splicing, leading to the production of two protein forms. Moreover, it reverses the extremely compact spike phenotype due to C3129T. Field experiments indicated that *Q*^*c1*^*-Q1* can increase wheat yield by simultaneously increasing the thousand-kernel weight and grain number per spike. Notably, the positive effect of *Q*^*c1*^*-Q1* on yield did not negatively affect grain quality. Furthermore, we developed a specific marker on the basis of G542A to accelerate the application of *Q*^*c1*^*-Q1* in wheat breeding programs.

**Conclusion:**

In this study, we developed a new approach to generating favorable *Q* alleles, while also producing germplasm potentially useful for breeding new wheat varieties with optimal grain production.

**Supplementary Information:**

The online version contains supplementary material available at 10.1186/s12870-026-08188-4.

## Background

Common wheat (*Triticum aestivum* L.) is one of the most widely cultivated and consumed staple food crops, providing approximately 18% of the calories and 19% of the proteins in the human diet [1]. Therefore, wheat cultivation is critical for global agricultural production and food security. Considering the increasing global population and decreasing arable land area, optimizing grain yield per unit area is a high priority for wheat breeding programs [2, 3]. Although there have been significant advancements in wheat production and research in the last two decades, the current rate of wheat yield increases cannot match the growing demand for wheat grains. Global climate change has exacerbated this issue because increasing temperatures, drought, and flooding are major threats to wheat production. Potential food shortages due to decreases in wheat production may adversely affect food security by 2050 [4–8]. Thus, novel genes associated with high wheat yields must be identified and used in wheat breeding programs.

Wheat yield is a complex trait determined by spike number per unit area, grain number per spike, and thousand-kernel weight (TKW). During wheat cultivation, increasing the seeding rate can effectively increase the spike number per unit area. Both TKW and grain number per spike are key factors for further improving wheat yield [9–11]. TKW is mainly determined by grain size (length and width), with relatively large grains generally conducive to seedling vigor and early growth, ultimately increasing yield [12–14]. Although genes regulating grain number per spike and TKW are key determinants of wheat yield, only a few genes or alleles are suitable for breeding [15–19].

In wheat, *Q* on the long arm of chromosome 5 A is one of the most important domestication genes and encodes a member of the APETALA2 (AP2) transcription factor family [20, 21]. *Q* influences many domestication-related traits, including threshability, spike morphology, plant height, rachis fragility, and flowering time [21–27]. The domesticated *Q* allele in modern common wheat cultivars arose from a spontaneous mutation to the microRNA172-binding region of the *q* allele in wild wheat during evolution [21]. A point mutation at the microRNA172-binding site (C3129T) of the *Q*^*c1*^ allele can further disrupt in vivo cleavage of *Q* transcripts by microRNA172, which explains why *Q*^*c1*^ is more highly expressed than the domesticated *Q* allele [28]. Overexpressing *Q*^*c1*^ significantly increases TKW, but results in compact spikes and plant dwarfism, thereby decreasing grain number per spike and yield. This yield penalty limits the utility of *Q*^*c1*^ for wheat breeding [27–29].

Alternative splicing, which may be useful for optimizing the *Q*^*c1*^ allele, is a common phenomenon in plants, with a crucial role in modulating gene expression during development and stress responses [30–33]. Alternative splicing produces multiple mRNAs from the same gene through the variable selection of splice sites during pre-mRNA splicing, which helps regulate post-transcriptional gene expression [34, 35]. There are various types of alternative splicing (e.g., skipped exon, intron retention, alternative 5′ splice site, alternative 3′ splice site, and mutually exclusive exon) [30, 36], among which intron retention occurs most frequently in plants. The regulation of alternative splicing depends on sequence elements in pre-mRNAs and interactions involving RNA-binding proteins. Alternative splicing leads to increased proteome diversity, while also regulating transcript levels and generating truncated proteins due to the introduction of premature stop codons in coding sequences [35].

Chemical mutagenesis, which is an efficient method for exploiting and improving genes controlling important traits, has been widely applied in common wheat breeding programs. To increase the utility of the *Q*^*c1*^ allele for wheat breeding, we produced an ethyl methanesulfonate (EMS)-mutagenized population of wheat *Q* gene mutant line *S-Cp1-1* (carrying the *Q*^*c1*^ allele) and identified a revertant *Q* gene mutant (*AS1* carrying the *Q*^*c1*^*-Q1* allele) with excellent agronomic traits. We also assessed the effect of *Q*^*c1*^*-Q1* on wheat grain yield and quality. The study findings may have implications for breeding novel wheat varieties with optimal grain production.

## Results

### Identification of the *Q*^*c1*^*-Q1* allele

**Fig. 1:**
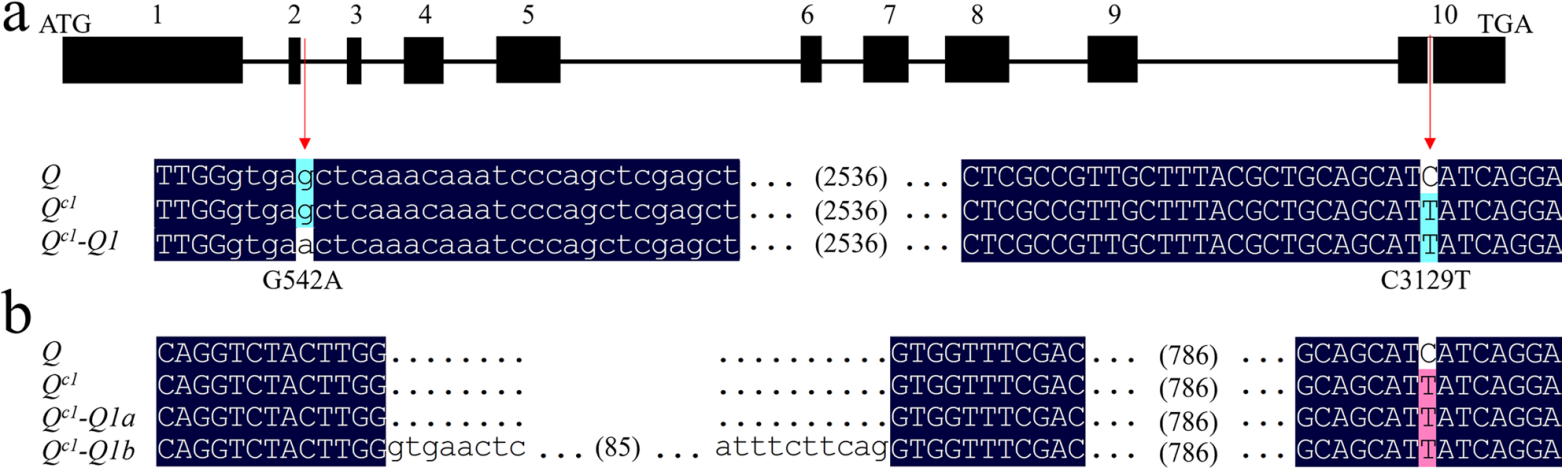
G542A results in the alternative splicing of the *Q*^*c1*^*-Q1* allele. **a** Distribution of two point mutations (G542A and C3129T) in the *Q* genomic DNA sequence. ‘ATG’ and ‘TGA’ are the start and stop codons of *Q*, respectively. The black box and thin line indicate exons (numbered 1–10) and introns, respectively. Uppercase letters indicate partial sequences of the second and tenth exons, whereas lowercase letters indicate partial sequences of the second intron. ‘… (2536) …’ indicates the 2,536 bases omitted between the second intron and tenth exon. **b** Alignment of partial cDNA sequences of the domesticated *Q* allele (Genebank No. KX580301), overexpressed *Q*^*c1*^ allele (KX580302), and two transcripts (*Q*^*c1*^*-Q1a* and *Q*^*c1*^*-Q1b*) of *Q*^*c1*^*-Q1* (PX626453). The second intron (indicated by lowercase letters) is retained in the *Q*^*c1*^*-Q1b* transcript. Uppercase letters indicate partial sequences of the second, third, and tenth exons. ‘… (85) …’ indicates the 85 bases omitted within the second intron. ‘… (786) …’ indicates the 786 bases omitted between the third and tenth exons. Two point mutations are indicated by red arrows

The genomic and cDNA sequences of *Q* were cloned from *AS1*. The subsequent sequencing and comparison with the *Q* genomic sequence in “Shumai482” (Genebank No. KX580301) detected two missense mutations in the *Q* sequence of *AS1* (Genebank No. PX626453). One missense mutation (in the initial codon) was a G-to-A substitution at nucleotide position 542 (G542A), which is located at the splice site of the second intron of *Q*. The other missense mutation (C3129T) in *Q* occurred at the microRNA172-binding site, similar to that in the reported *Q*^*c1*^ allele (Genebank No. KX580302; Figs. 1a and S1) [28]. The two transcripts for *Q*^*c1*^*-Q1* detected by cDNA cloning, namely *Q*^*c1*^*-Q1a*(1,344 bp) and *Q*^*c1*^*-Q1b* (1,447 bp), reflected an alternative splicing event (Figs. 1b and S2). The second intron of *Q*^*c1*^*-Q1* allele was retained in *Q*^*c1*^*-Q1b* (Fig. S2). Moreover, the cDNA sequences of *Q*^*c1*^*-Q1a* and *Q*^*c1*^ were same and therefore were predicted to produce the same functional protein, which was in contrast to the truncated protein predicted for *Q*^*c1*^*-Q1b* (Figs. S2 and S3).

### *Q*^*c1*^*-Q1* increased grain number per spike and thousand-kernel weight in the segregating population

To assess the effect of *Q*^*c1*^*-Q1* on grain yield and quality traits, *AS1* was backcrossed with “Shumai482” to obtain heterozygous BC_3_F_1_ and BC_3_F_3_ plants (Fig. S4), which were used to generate BC_3_F_2_ and BC_3_F_4_ segregating populations, respectively. The presence/absence of *Q*^*c1*^*-Q1* in each individual was determined by sequencing and examining the G542A mutation in the sequencing chromatogram. Under each growth condition, 30 plants homozygous for *Q*^*c1*^*-Q1* (SM482-Q^c1^-Q1) and 30 plants homozygous for domesticated *Q* (SM482-Q) were used to analyze agronomic traits.

**Fig. 2:**
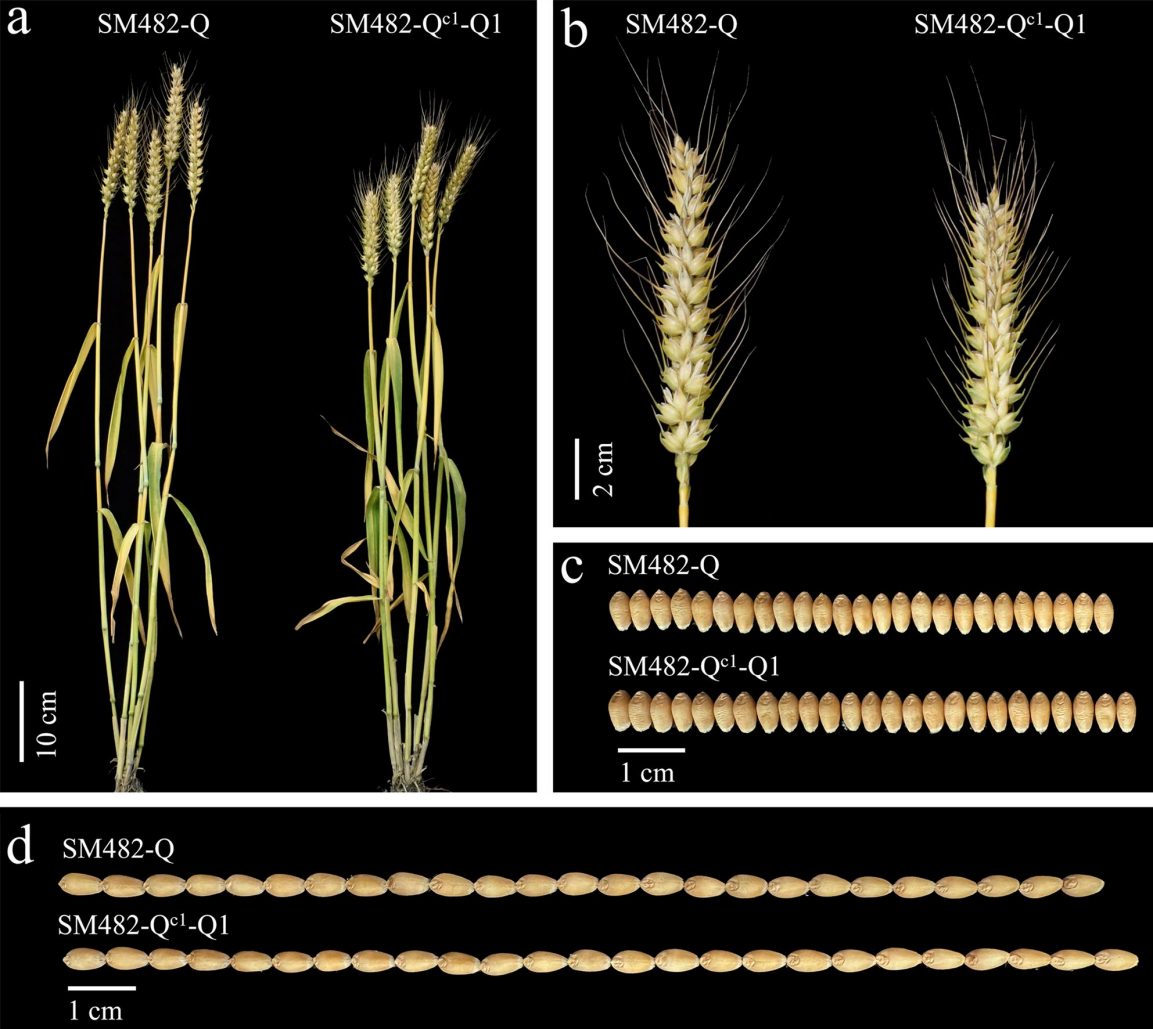
Comparison of the agronomic traits of SM482-Q^c1^-Q1 and SM482-Q plants under individual conditions. **a** SM482-Q^c1^-Q1 and SM482-Q plants at GS80. **b** SM482-Q^c1^-Q1 and SM482-Q spikes at GS80. SM482-Q^c1^-Q1 and SM482-Q mature grain width (**c**) and length (**d**)

Compared with SM482-Q plants in three environments, SM482-Q^c1^-Q1 plants were shorter (height decreased by 6.43–8.02 cm), with shorter spikes (decreased by 1.77–2.15 cm), more grains per spike (increased by 4.03–5.83), higher TKW (increased by 1.91–3.65 g), longer grains (increased by 0.19–0.24 mm), and wider grains (increased by 0.13–0.17 mm), but they were similar in terms of spikelet number per spike, productive tiller number, and grain protein content (GPC) (Fig. 2; Table 1).

**Table 1:** Comparison of the agronomic traits and GPC of SM482-Q^c1^-Q1 and SM482-Q under individual conditions

Traits	Genotype	2021–2022 WJ	2023–2024 WJ	2023–2024 CZ	E	G	E × G
Plant height (cm)	SM482-Q	76.16 ± 2.76	86.68 ± 2.17	83.67 ± 3.20	242.09**	281.64**	1.15
	SM482-Q^c1^-Q1	68.14 ± 3.04**	80.25 ± 2.44**	76.43 ± 3.50**			
Spike length (cm)	SM482-Q	14.81 ± 1.14	12.72 ± 0.66	12.48 ± 1.02	124.97**	216.00**	0.68
	SM482-Q^c1^-Q1	12.84 ± 0.99**	10.95 ± 0.77**	10.33 ± 0.66**			
Spikelet number per spike	SM482-Q	22.27 ± 1.05	24.03 ± 0.89	22.67 ± 1.06	39.28**	8.23**	0.29
	SM482-Q^c1^-Q1	21.77 ± 1.17	23.43 ± 1.01*	22.37 ± 1.33			
Grain number per spike	SM482-Q	78.47 ± 8.59	46.40 ± 4.10	47.87 ± 3.69	511.39**	30.97**	0.40
	SM482-Q^c1^-Q1	84.27 ± 7.84**	50.43 ± 6.29**	53.70 ± 5.70**			
Grain length (mm)	SM482-Q	6.35 ± 0.19	6.00 ± 0.16	6.23 ± 0.20	45.09**	56.76**	0.25
	SM482-Q^c1^-Q1	6.54 ± 0.23**	6.24 ± 0.17**	6.44 ± 0.17**			
Grain width (mm)	SM482-Q	3.24 ± 0.12	3.00 ± 0.08	3.09 ± 0.09	84.69**	72.08**	0.72
	SM482-Q^c1^-Q1	3.41 ± 0.19**	3.13 ± 0.07**	3.22 ± 0.07**			
Thousand kernel weight (g)	SM482-Q	52.21 ± 2.77	52.66 ± 2.03	51.00 ± 1.54	5.88**	68.29**	2.17
	SM482-Q^c1^-Q1	54.12 ± 3.19*	55.84 ± 2.45**	54.65 ± 1.80**			
Tiller number	SM482-Q	5.37 ± 1.45	4.50 ± 1.50	4.83 ± 1.15	7.00**	6.24*	0.19
	SM482-Q^c1^-Q1	5.83 ± 1.42	4.87 ± 1.22	5.50 ± 1.28*			
Grain protein content (%, dry weight)	SM482-Q	10.65 ± 0.96	13.64 ± 0.80	13.45 ± 0.86	206.09**	0.41	2.79
	SM482-Q^c1^-Q1	10.97 ± 0.80	13.36 ± 0.77	13.18 ± 0.66			

Data are presented as the mean ± standard deviation

*E* Environment, *G* Genotype, *E × G* Interaction between the environment and genotype, *WJ* Wenjiang farm, *CZ* Chongzhou farm

**, *P* < 0.01; *, *P* < 0.05

### *Q*^*c1*^*-Q1* improved yield without a quality penalty under wheat production conditions

SM482-Q^c1^-Q1 and SM482-Q (control) lines (Fig. S4) were used to assess the effects of *Q*^*c1*^*-Q1* on agronomic traits as well as yield and quality characteristics in three environments under wheat production conditions. TKW (increased by 1.98–2.85 g), grain length (increased by 0.16–0.33 mm), grain width (increased by 0.13–0.21 mm), grain number per spike (increased by 3.07–3.50), and yield (increased by 0.05–0.07 kg/m^2^) were greater for SM482-Q^c1^-Q1 than for SM482-Q, whereas the opposite trend was detected for plant height (decreased by 3.20–6.27 cm) and spike length (decreased by 1.71–1.86 cm). By contrast, spikelet number per spike and spike number per unit area did not differ significantly between SM482-Q^c1^-Q1 and SM482-Q (Table 2).

**Table 2:** Comparison of the agronomic traits of SM482-Q^c1^-Q1 and SM482-Q under wheat production conditions

Traits	Genotype	2022-2023WJ	2023-2024WJ	2023-2024CZ	E	G	E × G
Plant height (cm)	SM482-Q	86.82 ± 2.06	87.50 ± 2.09	84.35 ± 2.89	20.56**	140.36**	6.402**
	SM482-Q^c1^-Q1	80.55 ± 2.13**	83.73 ± 2.29**	81.15 ± 3.26**			
Spike length (cm)	SM482-Q	12.71 ± 0.70	11.30 ± 0.65	11.72 ± 0.73	70.29**	297.26**	0.21
	SM482-Q^c1^-Q1	10.97 ± 0.92**	9.44 ± 0.49**	10.01 ± 0.57**			
Spikelet number per spike	SM482-Q	23.50 ± 1.14	21.47 ± 1.46	22.03 ± 1.19	44.79**	2.91	0.01
	SM482-Q^c1^-Q1	23.20 ± 1.06	21.13 ± 1.41	21.73 ± 1.01			
Grain number per spike	SM482-Q	52.83 ± 4.68	33.63 ± 4.03	41.13 ± 5.26	215.42**	18.58**	0.03
	SM482-Q^c1^-Q1	56.17 ± 7.09*	36.70 ± 4.41**	44.63 ± 4.77**			
Grain length (mm)	SM482-Q	6.86 ± 0.26	6.22 ± 0.08	6.20 ± 0.15	70.91**	23.95**	0.89
	SM482-Q^c1^-Q1	7.16 ± 0.08*	6.38 ± 0.10*	6.53 ± 0.15**			
Grain width (mm)	SM482-Q	3.56 ± 0.09	3.02 ± 0.04	3.04 ± 0.08	162.29**	36.98**	0.62
	SM482-Q^c1^-Q1	3.73 ± 0.06*	3.15 ± 0.07**	3.25 ± 0.10**			
Thousand kernel weight (g)	SM482-Q	48.89 ± 0.43	47.27 ± 0.83	49.37 ± 0.51	18.50**	84.04**	0.96
	SM482-Q^c1^-Q1	50.87 ± 1.30*	50.12 ± 0.59**	52.07 ± 0.45**			
Spike number per unit area	SM482-Q	390.80 ± 14.24	353.20 ± 22.49	344.80 ± 14.24	18.70**	0.29	0.04
	SM482-Q^c1^-Q1	397.20 ± 16.13	355.60 ± 27.65	347.00 ± 13.36			
Grain yield (kg/m^2^)	SM482-Q	0.78 ± 0.02	0.46 ± 0.02	0.48 ± 0.02	679.07**	58.44**	0.75
	SM482-Q^c1^-Q1	0.85 ± 0.02**	0.51 ± 0.01**	0.54 ± 0.04*			

Data are presented as the mean ± standard deviation

*E* Environment, *G* Genotype, *E × G* Interaction between the environment and genotype, *WJ* Wenjiang farm, *CZ* Chongzhou farm

**, *P* < 0.01; *, *P* < 0.05

In terms of quality-related characteristics, volume weight was lower (decreased by 6.50–10.94 g) for SM482-Q^c1^-Q1 than for SM482-Q in three environments. There were no significant differences in GPC, Zeleny sedimentation value, wet gluten content, water absorption, dough stability time, loaf volume, biscuit diameter, biscuit thickness, and spread ratio between SM482-Q^c1^-Q1 and SM482-Q in three environments (Fig. S5 and Table 3), with the exception of the Zeleny sedimentation value and dough stability time, which were significantly higher for SM482-Q^c1^-Q1 than for SM482-Q in 2022–2023 in Wenjiang.

**Table 3:** Comparison of the processing quality parameters of SM482-Q^c1^-Q1 and SM482-Q under wheat production conditions

Traits	Genotype	2022-2023WJ	2023-2024WJ	2023-2024CZ	E	G	E × G
Grain protein content (%, dry weight)	SM482-Q	14.23 ± 0.41	14.51 ± 0.15	13.55 ± 0.42	17.68**	1.13	0.05
	SM482-Q^c1^-Q1	14.04 ± 0.49	14.37 ± 0.26	13.46 ± 0.31			
Zeleny sedimentation value (mL)	SM482-Q	19.80 ± 0.45	22.20 ± 0.76	20.40 ± 1.39	10.61**	3.77	0.04
	SM482-Q^c1^-Q1	20.80 ± 0.84*	23.04 ± 1.49	21.10 ± 1.71			
Wet gluten content (%)	SM482-Q	24.44 ± 0.79	31.58 ± 1.16	28.17 ± 1.55	104.68**	0.65	0.11
	SM482-Q^c1^-Q1	24.28 ± 0.95	31.36 ± 0.93	27.58 ± 1.06			
Volume weight (g)	SM482-Q	842.78 ± 2.05	788.16 ± 4.28	775.26 ± 4.52	531.26**	24.35**	0.75
	SM482-Q^c1^-Q1	833.15 ± 6.12*	777.22 ± 6.25*	768.76 ± 4.21*			
Water absorption (%)	SM482-Q	48.74 ± 0.29	58.34 ± 0.50	55.86 ± 1.15	632.59**	0.42	1.27
	SM482-Q^c1^-Q1	48.40 ± 0.29	58.56 ± 0.76	56.44 ± 0.48			
Dough stability time (min)	SM482-Q	5.72 ± 0.71	3.06 ± 0.15	1.99 ± 0.12	140.67**	7.74*	5.16*
	SM482-Q^c1^-Q1	7.35 ± 1.27*	3.50 ± 0.40	1.84 ± 0.30			
Loaf volume (mL)	SM482-Q	227.00 ± 7.45	244.00 ± 8.83	240.20 ± 6.30	18.00**	2.83	0.77
	SM482-Q^c1^-Q1	228.60 ± 10.78	254.00 ± 5.24	243.40 ± 8.38			
Biscuit diameter (mm)	SM482-Q	103.54 ± 1.22	105.26 ± 2.65	105.07 ± 2.16	1.11	4.12	0.32
	SM482-Q^c1^-Q1	102.76 ± 2.16	103.75 ± 2.08	102.83 ± 1.66			
Biscuit thickness (mm)	SM482-Q	8.76 ± 0.34	9.25 ± 0.27	9.05 ± 0.54	6.68**	0.84	0.19
	SM482-Q^c1^-Q1	8.84 ± 0.34	9.48 ± 0.34	9.10 ± 0.10			
Spread ratio (diameter/thickness)	SM482-Q	11.83 ± 0.52	11.39 ± 0.60	11.65 ± 0.94	1.98	1.89	0.10
	SM482-Q^c1^-Q1	11.65 ± 0.70	10.97 ± 0.57	11.30 ± 0.27			

Data are presented as the mean ± standard deviation

*E* Environment, *G* Genotype, *E × G* Interaction between the environment and genotype, *WJ* Wenjiang farm, *CZ* Chongzhou farm

**, *P* < 0.01; *, *P* < 0.05

### Development of a specific marker for *Q*^*c1*^*-Q1*

**Fig. 3:**
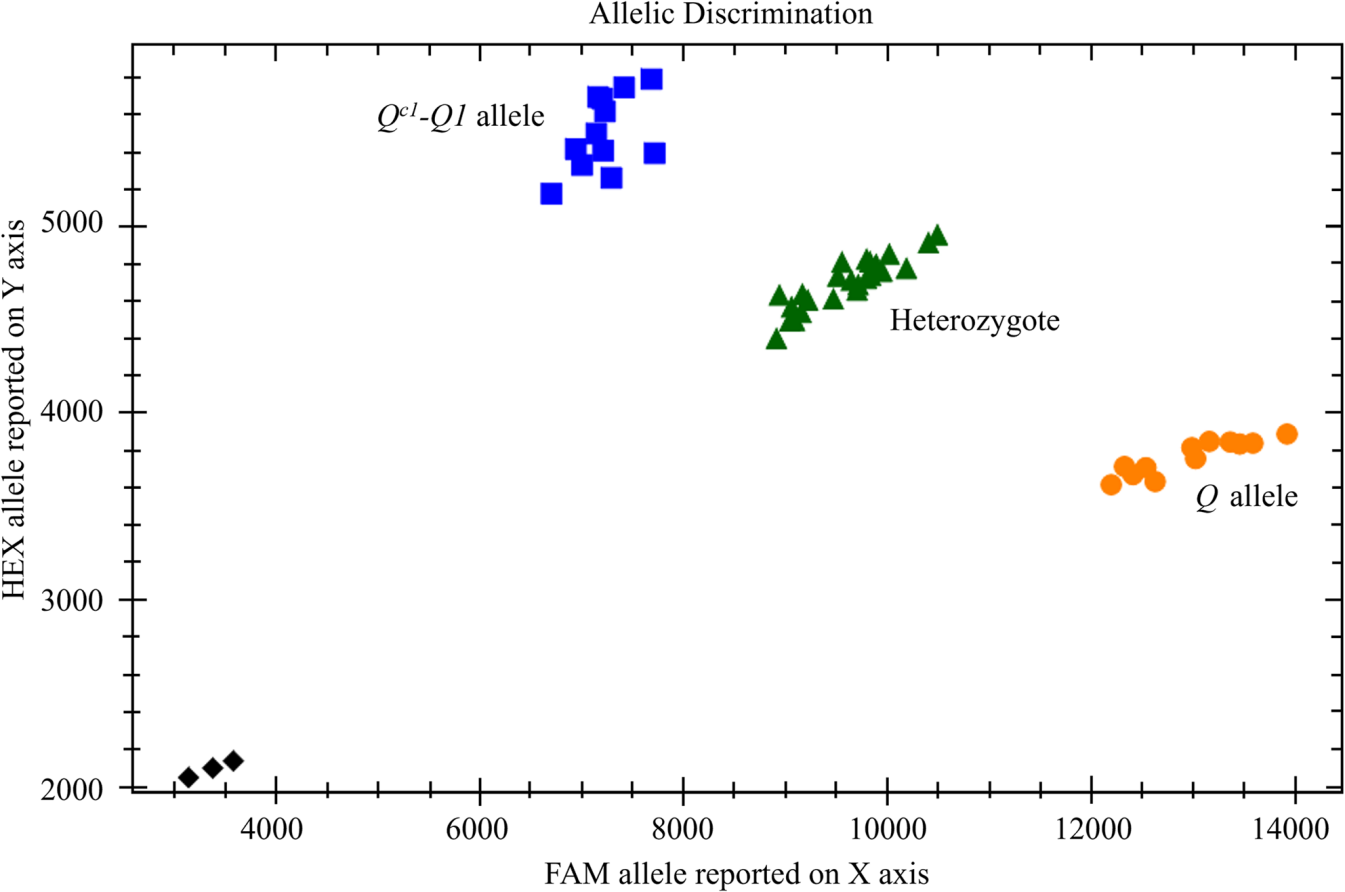
Utility of KASP marker *KASP-Q*^*c1*^*-Q1* for detecting *Q*^*c1*^*-Q1* in BC_3_F_2_ and BC_3_F_4_ segregating populations. Blue squares, green rectangles, and orange circles represent samples homozygous for *Q*^*c1*^*-Q1*, samples with both *Q*^*c1*^*-Q1* and the domesticated *Q* allele, and samples homozygous for *Q*, respectively

To efficiently detect *Q*^*c1*^*-Q1*, a kompetitive allele-specific PCR (KASP) marker was designed on the basis of G542A. Primers KASP-Q^c1^-Q1-F1 and KASP-Q^c1^-Q1-F2 (Table S1) were designed with standard FAM and HEX tails at the 5′ end, respectively, and with the target G and A bases at the 3′ end, respectively. The utility of the developed KASP marker was assessed in the BC_3_F_2_ and BC_3_F_4_ segregating populations (Fig. S4), with *AS1* and “Shumai482” used as controls. The detection results obtained for the KASP marker were consistent with those obtained by sequencing (Fig. 3).

### Expression analysis

**Fig. 4:**
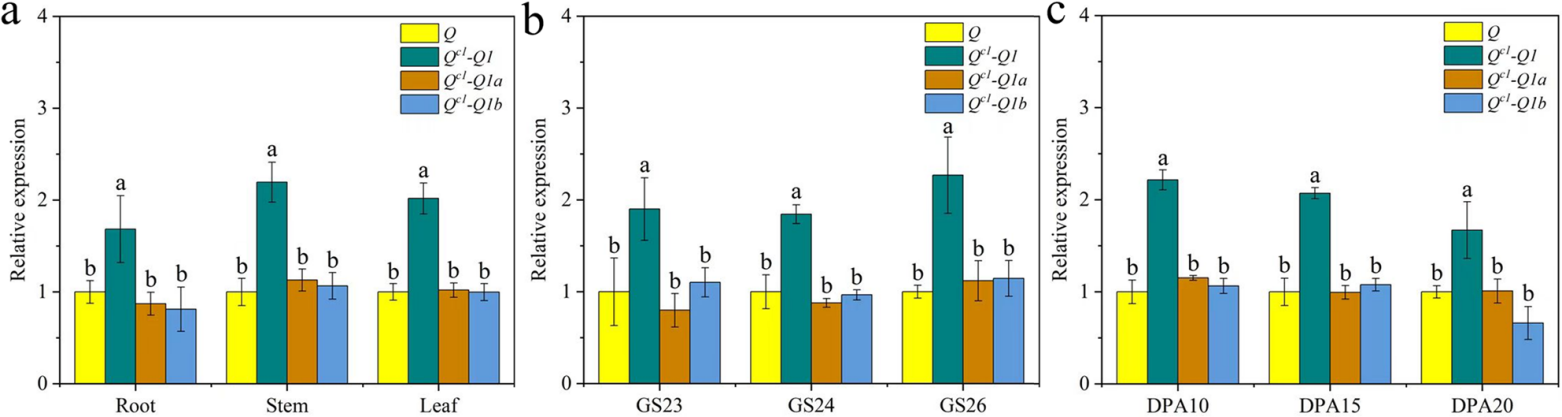
Domesticated *Q* allele and *Q*^*c1*^*-Q1* allele expression levels. Comparison of the expression levels of domesticated *Q*, *Q*^*c1*^*-Q1*, *Q*^*c1*^*-Q1a*, and *Q*^*c1*^*-Q1b* in roots, stems, and leaves at GS20 (**a**), in developing spikes at GS23, GS24, and GS26 (**b**), and in young grains at 10, 15, and 20 DPA (**c**) of SM482-Q^c1^-Q1 and SM482-Q. Expression levels were determined by reverse transcription PCR. Different lowercase letters above columns indicate significant differences (*P* < 0.05) for each sample type

Reverse transcription (RT) PCR was performed to compare *Q* expression in SM482-Q^c1^-Q1 and SM482-Q (Fig. S6). In roots, stems, and leaves at GS20 (a decimal code system for wheat development) [37], and in developing spikes (GS23, GS24, and GS26), and in young grains [10, 15, and 20 days post anthesis (DPA)], the *Q*^*c1*^*-Q1* expression level in SM482-Q^c1^-Q1 was significantly higher than the domesticated *Q* expression level in SM482-Q. Notably, *Q*^*c1*^*-Q1a* and *Q*^*c1*^*-Q1b* transcript levels in SM482-Q^c1^-Q1 were similar to the domesticated *Q* transcript level in SM482-Q (Fig. 4).

### Q^c1^-Q1a and Q^c1^-Q1b altered nuclear transcriptional repression

**Fig. 5:**
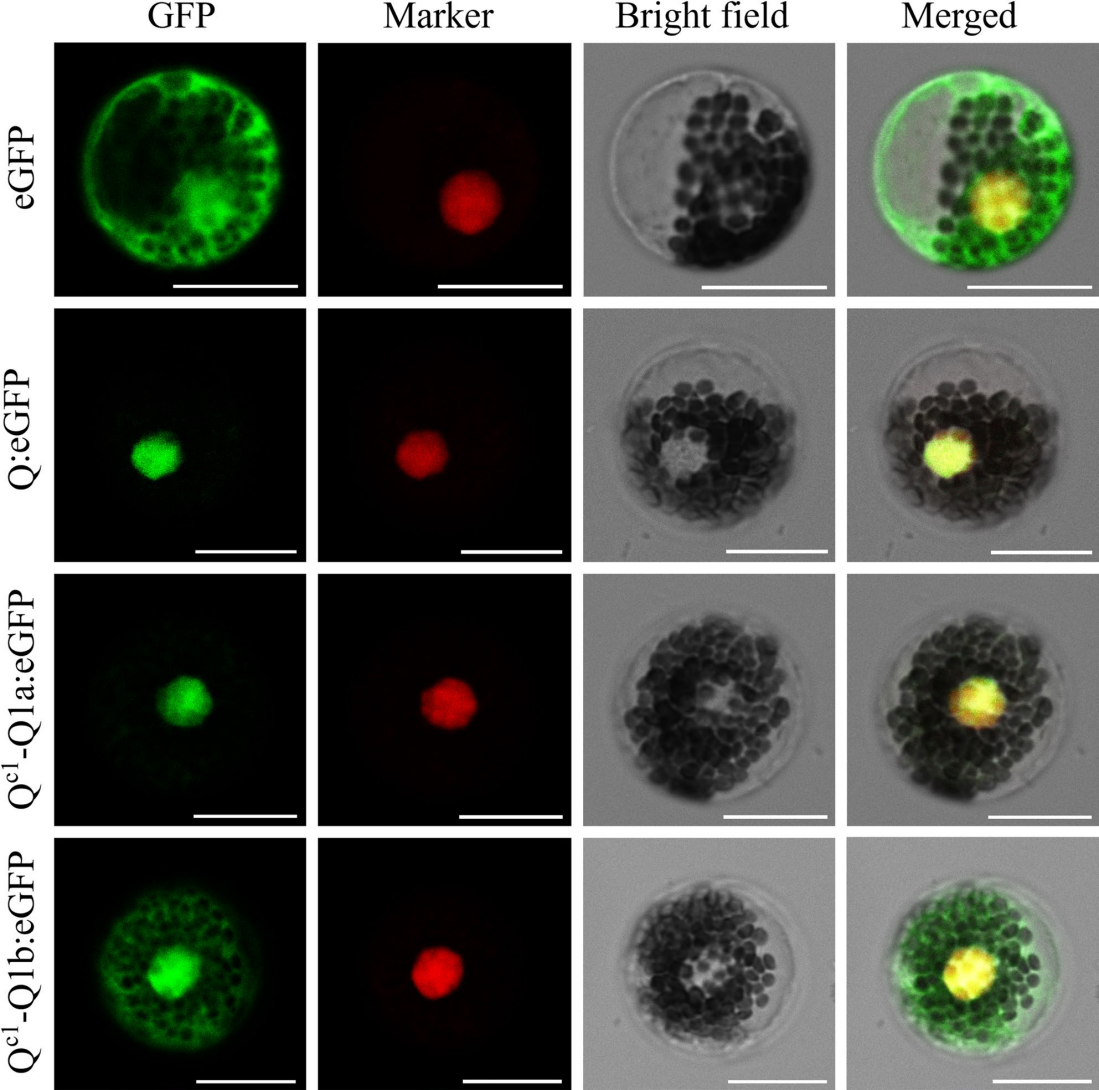
Localization of Q^c1^-Q1a and Q^c1^-Q1b proteins in wheat mesophyll protoplasts. eGFP, Q:eGFP, Q^c1^-Q1a: eGFP, and Q^c1^-Q1b: eGFP represent pJIT163-eGFP, pJIT163-Q-eGFP, pJIT163-Q^c1^-Q1a-eGFP, and pJIT163-Q^c1^-Q1b-eGFP vectors, respectively. Scale bar, 20 μm

To determine the subcellular localization of Q^c1^-Q1a and Q^c1^-Q1b, wheat mesophyll protoplasts were transformed with recombinant vectors pJIT163-Q^c1^-Q1a-eGFP and pJIT163-Q^c1^-Q1b-eGFP. Microscopic examinations indicated that Q^c1^-Q1a and Q were distributed in the nucleus, whereas Q^c1^-Q1b was distributed in the nucleus and cytoplasm (Fig. 5).

We also determined the transcriptional activity of Q^c1^-Q1a and Q^c1^-Q1b using a transient dual-luciferase expression system involving *Nicotiana benthamiana* leaves. Under our experimental conditions, *Q* expression significantly decreased the firefly luciferase (LUC):*Renilla* luciferase (REN) activity ratio (compared with the control), indicating that Q is a transcriptional repressor, which is in accordance with the findings of an earlier study [38]. The LUC: REN ratio of Q^c1^-Q1a was higher than that of Q, but lower than that of the control, reflecting a partial decrease in transcriptional repression. The LUC: REN ratio of Q^c1^-Q1b was higher than that of Q, but similar to that of the control, reflecting the elimination of transcriptional repression (Fig. S7).

### Potential use of *Q*^*c1*^*-Q1* for breeding

To assess the utility of *Q*^*c1*^*-Q1* for breeding, *AS1* was used as the male parent for a cross with common wheat cultivar “Chuanmai620”. Three superior F_4_ lines carrying *Q*^*c1*^*-Q1* were selected. Compared with “Chuanmai620”, these three superior lines produced more grains per spike, with longer and wider grains and a higher TKW. Compared with *AS1*, the three superior lines produced more grains per spike, with wider, but shorter, grains and a similar TKW (Fig. S8 and Table S2).

## Discussion

### G542A leads to alternative splicing of *Q*^*c1*^*-Q1*

RNA splicing, which involves the removal of introns from pre-mRNA, is an important process during gene expression in eukaryotes. Some pre-mRNAs have more than one splicing pattern (i.e., alternative splicing). Spliceosome assembly during intron removal and ligation of exons is directed by pre-mRNA sequence features [35, 39]. On the basis of earlier research, pre-mRNAs contain 5′ splice site (splice donor), 3′ splice site (splice acceptor), branch point, polypyrimidine tract, enhancer, and suppressor sequences [39]. The 5′ splice site at the 5′ end of the intron includes a GT dinucleotide at the exon/intron junction, while the 3′ splice site at the 3′ end of the intron contains the following three conserved sequence elements: branch point followed by a polypyrimidine tract and a terminal AG. Correct pre-mRNA splicing requires the recognition of splice sites at exon–intron borders [36, 40]. Single-base mutations at the splice sites of the rice *waxy* gene and wheat *Q* gene disrupt pre-mRNA processing and prevent intron removal [21, 41, 42]. A splice acceptor site mutation (G2373A) results in the incorrect splicing of the *TaGW2-A1* transcript, leading to two alternative protein forms and an increase in TKW [11, 43]. A single point mutation at the splice site of the wheat gene encoding starch synthase 2a results in abnormal RNA splicing, which decreases the total starch content, but increases the resistant starch content [44]. In the current study, the point mutation G542A in *Q*^*c1*^*-Q1* was located near the GT dinucleotide at the boundary of the second exon and second intron of *Q* (Figs. 1 and S1), thereby leading to the alternative splicing of *Q*^*c1*^*-Q1*.

### Alternative splicing is a new way to generate favorable *Q* alleles for breeding

MicroRNAs regulate gene expression mainly by eliminating DNA, cleaving mRNA, and repressing translation [45–47]. Base pairing between microRNAs and their target sites is crucial for mRNA cleavage [48]. The interaction between microRNA172 and its *AP2* or *AP2*-like targets is highly conserved, with important implications for plant developmental regulation [49, 50]. In barley, base changes at the microRNA172-binding site of *HvAP2* can enhance transcription [51, 52]. The *Q* gene has a microRNA172-binding site in its tenth exon. Point mutations at the microRNA172-binding site can adversely affect *in vivo* cleavage by microRNA172, leading to *Q* overexpression [21, 28, 46]. Notably, *Q*^*c1*^ overexpression can significantly increase TKW, but the associated production of extremely compact spikes as well as plant dwarfism lead to decreases in the number of grains per spike and yield.

As important parts of the Q protein, the first and second AP2 domains are critical for DNA binding and for physical interactions with other proteins. Missense mutations in the *Q* sequence encoding AP2 domains can decrease spike density and increase plant height [21, 24, 29]. Accordingly, we introduced a missense mutation to the sequence encoding the second AP2 domain in the overexpressed *Q*^*c1*^ allele. The expression of the resulting *Q*^*c1*^*-N8* allele reversed the mutant phenotypes (i.e., extremely compact spike and dwarfism) caused by *Q*^*c1*^ expression. Moreover, it positively affected yield (i.e., increased TKW and grain number per spike) [29], reflecting the benefits of optimizing *Q* sequences.

We herein describe a new way to optimize *Q* gene sequences. To prevent the development of compact spikes and plant dwarfism, a point mutation (G542A) was introduced into the 5′ splice site of the second intron of *Q*^*c1*^ (Fig. S1), which resulted in the alternative splicing of *Q* transcripts and generated the *Q*^*c1*^*-Q1* allele. Two transcripts were detected for *Q*^*c1*^*-Q1* (*Q*^*c1*^*-Q1a* and *Q*^*c1*^*-Q1b*) (Figs. 1 and S2). Alternative splicing can regulate mRNA levels via the production of mRNA isoforms containing premature termination codons [34, 43]. *Q*^*c1*^*-Q1a* was predicted to produce the same functional protein as *Q*^*c1*^, while *Q*^*c1*^*-Q1b* was predicted to encode a truncated protein missing an AP2 domain (Fig. S3). Q reportedly functions as a transcriptional repressor under certain conditions [38]. Notably, transcriptional repression effects were lower for Q^c1^-Q1a and Q^c1^-Q1b than for Q (Fig. S7). Interestingly, RT-PCR results indicated that *Q*^*c1*^*-Q1a* and *Q*^*c1*^*-Q1b* were expressed at similar levels (Fig. 4). The *Q* gene copy number is positively and negatively correlated with spike density and plant height, respectively, with the addition of another *Q* copy in the wheat genome resulting in a plant phenotype similar to that induced by the *Q*^*c1*^ allele [53, 54]. *Q*^*c1*^*-Q1* expression decreased the production of functional Q proteins that regulate wheat growth and development. Under both individual and wheat production conditions, *Q*^*c1*^*-Q1* expression significantly improved yield by increasing TKW and grain number per spike (Tables 1 and 2). Furthermore, the primary use of *Q*^*c1*^*-Q1* in southwestern China reflects its potential utility for wheat breeding (Fig. S8 and Table S2). Therefore, in addition to introducing missense mutations in AP2 domain-encoding sequences, alternative splicing represents a new way to optimize *Q* gene sequences and generate favorable *Q* alleles relevant to wheat breeding.

### *Q*^*c1*^*-Q1* balances yield and quality

Wheat processing quality is the major determinant of consumer preferences for specific wheat cultivars. Moreover, wheat yield increases are critical for ensuring food security [2, 55, 56]. GPC is a key indicator of wheat processing quality. Optimizing GPC through genetic modifications is an important objective of wheat breeding programs because of the potential economic benefits associated with decreasing the frequency of nitrogen fertilizer applications and improving human health via the production of nutrient-rich wheat grains [57, 58]. However, breeding wheat cultivars with high yields and high-quality grains using conventional breeding techniques is challenging because of the negative correlation between GPC and yield [59, 60]. There is still a lack of genes useful for enhancing both GPC and yield, although promising results have been reported for *Q* [28, 29]. Specifically, *Q* belongs to a regulatory network that modulates photosynthesis as well as carbon and nitrogen metabolism [61]. In the present study, we identified the *Q*^*c1*^*-Q1* allele, which can increase wheat yield without adversely affecting grain quality, indicating that it may be useful for breeding wheat varieties with improved grain production.

## Materials and methods

### Plant materials and growth conditions

Mature seeds of the *Q* gene mutant line *S-Cp1-1* (carrying the *Q*^*c1*^ allele; with extremely compact spike and dwarf plant height [28]) were treated with 0.4% EMS (Sigma-Aldrich, St Louis, MO, USA). Seeds from the leading spikes of M_1_ plants were harvested and sown to generate the M_2_ population. The *Q* gene mutant *AS1* (carrying the *Q*^*c1*^*-Q1* allele; with normal spike morphology and plant height) was isolated from the M_2_ population. *S-Cp1-1* was previously isolated from an EMS-mutagenized population of common wheat cultivar “Shumai482” (carrying the domesticated *Q* allele) [28]. To purify the genetic background, *AS1* was backcrossed with “Shumai482” to generate BC_3_F_2_ and BC_3_F_4_ segregating populations (derived from a single heterozygous BC_3_F_1_ plant and a single heterozygous BC_3_F_3_ plant, respectively). Segregating populations were used to assess the effect of *Q*^*c1*^*-Q1* under individual conditions. Subsequently, SM482-Q^c1^-Q1 (carrying the *Q*^*c1*^*-Q1* allele; BC_3_F_4_ generation) and its control line SM482-Q (carrying the domesticated *Q* allele) were obtained (Fig. S4) and used to examine the effect of *Q*^*c1*^*-Q1* under wheat production conditions.

To evaluate the effect of *Q*^*c1*^*-Q1* under individual conditions, seeds collected from heterozygous BC_3_F_1_ and BC_3_F_3_ plants were sown in fields (row spacing of 20 cm × 10 cm) at the experimental farms (Wenjiang and Chongzhou) of Sichuan Agricultural University during the 2021–2022 and 2023–2024 wheat growing seasons. Genomic DNA was extracted from individual plants for the subsequent genotyping of *Q*^*c1*^*-Q1*.

To analyze the effect of *Q*^*c1*^*-Q1* under wheat production conditions, SM482-Q^c1^-Q1 and SM482-Q lines were grown at the Wenjiang and Chongzhou experimental farms during the 2022–2023 and 2023–2024 wheat growing seasons. Five replicates per line were arranged in a randomized block design. Each replicate was grown in a 2 m × 2 m plot, with 20 cm between rows and 100 seeds per row. A nitrogen: phosphorous: potassium (15:15:15) compound fertilizer was applied (600 kg per hectare) before sowing. Pest control and weed management practices were in accordance with those used in local fields.

### Nucleotide extraction

Genomic DNA was extracted from fresh leaves at GS13 (decimal code system for wheat development stages) using cetyltrimethylammonium bromide as previously described [37, 62]. In addition, total RNA was extracted and then reverse transcribed to cDNA using a Plant RNA Extraction kit (Biofit, Chengdu, China) and a HiScript III 1 st Strand cDNA Synthesis Kit (Vazyme, Nanjing, China), respectively.

### Gene cloning

*Q*^*c1*^*-Q1* cDNA and genomic DNA sequences were cloned and sequenced as previously described [29]. Primers (Table S1) were designed using Premier 5.0 (Premier Biosoft, San Francisco, CA, USA). PCR amplifications were performed in a 50 µL reaction volume using Phanta Mix Super-Fidelity DNA polymerase P505 (Vazyme). PCR mixtures consisted of 100 ng genomic DNA or cDNA template, 100 µM each dNTP, 20 pmol each primer, 1 U DNA polymerase, and 25 µL 2× buffer (with 4 mM Mg^2+^). The PCR program, which was completed using a Mastercycler Pro thermal cycler (Eppendorf, Hamburg, Germany), was as follows: 95 °C for 5 min; 35 cycles of 95 °C for 30 s, 60 °C for 1 min, and 72 °C for 1 min; 72 °C for 10 min. PCR products were separated on a 1.5% agarose gel. Target fragments were purified using a FastPure Gel DNA Extraction Mini Kit (Vazyme) and then inserted into the pCE2 TA/Blunt-Zero vector using a 5 min TA/Blunt-Zero Cloning Kit (Vazyme). Positive colonies were sequenced by Sangon Biotech (Chengdu, China). Cloning and sequencing experiments were repeated three times. Sequences were analyzed using DNAMAN (version 8) (Lynnon Biosoft, San Ramon, CA, USA).

### Genotyping of *Q*^*c1*^*-Q1*

Genomic DNA was extracted from individual plants in the BC_3_F_2_ and BC_3_F_4_ populations (described above) and then used as the template for a PCR amplification involving primers AP2startF and AP2.8R (Table S1). The PCR amplification was performed as described above. PCR products were sequenced by Sangon Biotech. The presence/absence of *Q*^*c1*^*-Q1* was determined by examining the G542A mutation in the sequencing chromatogram.

### Development of a KASP marker for *Q*^*c1*^*-Q1*

The G542A mutation in the second intron of the *Q*^*c1*^*-Q1* allele was converted to a KASP marker (*KASP-Q*^*c1*^*-Q1*; http://www.polymarker.info) [63]. The utility of this marker was tested using the BC_3_F_2_ and BC_3_F_4_ populations as described above. Both “Shumai482” and *AS1* served as controls for three biological replicates. HiGeno 2× Probe Mix B (JasonGen, Beijing, China) and a CFX 96 Real-Time System (Bio-Rad, Hercules, CA, USA) were used according to manufacturer instructions.

### Examination of agronomic traits and processing quality

Under both individual and wheat production conditions, SM482-Q^c1^-Q1 and SM482-Q plants at GS87 were compared in terms of their agronomic traits, including plant height (cm), spike length (cm), spikelet number per spike, grain number per spike, spike number per unit area (wheat production conditions only), and effective tiller number (individual conditions only). Additionally, an SC-G automatic seed analyzer (Wanshen, Hangzhou, China) was used to measure TKW (g), grain length (mm), and grain width (mm) of sun-dried mature seeds.

Mature grain samples were stored at room temperature for 2 months before being milled using a CD1 Laboratory Mill (Chopin, Villeneuve-la-Garenne Cedex, France) for the subsequent analysis of the wet gluten content, GPC (dry weight), Zeleny sedimentation value, dough rheological properties, and loaf and biscuit quality parameters as previously described [59, 64].

### Reverse transcription PCR

SM482-Q^c1^-Q1 and SM482-Q plants were grown in a glasshouse under a 12-h light (25 °C):12-h dark (20 °C) cycle. Root, stem, and leaf samples were collected at GS20 for an analysis of gene expression. Spike samples were collected at GS23, GS24, and GS26 and developing grain samples were collected at 10, 15, and 20 DPA from plants cultivated in Wenjiang during the 2022–2023 wheat growing season. Three biological replicates were prepared. Total RNA was extracted as described above, after which *Q* expression levels were analyzed via RT-PCR. A housekeeping gene encoding a scaffold-associated region DNA-binding protein (NCBI UniGene: *Ta.14126*; Genebank No. BE429982; https://www.ncbi.nlm.nih.gov/) was used to normalize gene expression data [65]. Details regarding RT-PCR primers are provided in Table S1. PCR conditions were as follows: 94 °C for 5 min; 32 cycles of 94 °C for 15 s, 60 °C for 15 s, and 72 °C for 15 s; and 72 °C for 5 min. The PCR product (5 µL) of each sample was electrophoresed (three technical replicates) in a 1.5% agarose gel, which was stained with ethidium bromide (1 µg/mL), and visualized using a Gel Dox XR + gel imaging analysis system (Bio-Rad). The color of raw agarose gel images was adjusted so that bands appeared black in a white background. Bands were analyzed to determine the gray value of each PCR product using the quantity tools of ImageJ software [26, 66].

### Subcellular localization

Coding sequences of the domesticated *Q* allele and the two transcripts of *Q*^*c1*^*-Q1* (*Q*^*c1*^*-Q1a* and *Q*^*c1*^*-Q1b*) were fused with the green fluorescent protein gene (*GFP*) in the pJIT163-eGFP vector. Primer details are provided in Table S1. The resulting pJIT163-Q-eGFP, pJIT163-Q^c1^-Q1a-eGFP, and pJIT163-Q^c1^-Q1b-eGFP recombinant vectors were inserted into wheat mesophyll protoplasts via PEG-mediated transfection [67]. After a 24-h incubation at 25 °C in darkness, transfected protoplasts were examined using a STELLARIS STED/EM CPD300 confocal microscope (Leica, Wetzlar, Germany).

### Luciferase-based transient transcriptional assay

Two reporter constructs were produced as previously described [68]. Specifically, 5×UAS-mini35S::LUC contained the firefly luciferase gene (*LUC*) ligated to a GAL4 upstream activation sequence (UAS) and a minimal 35S promoter (mini35S). The other reporter construct contained the *Renilla* luciferase gene (*REN*) under the control of the 35S promoter as an internal reference. Coding sequences of the domesticated *Q* allele, *VP16* gene, and two transcripts of *Q*^*c1*^*-Q1* (*Q*^*c1*^*-Q1a* and *Q*^*c1*^*-Q1b*) were ligated to the GAL4 DNA-binding domain (GAL4-BD) sequence to generate effector constructs under the control of the 35S promoter. All constructs were incorporated into the pCAMBIA1300 vector, with the resulting recombinant vectors inserted into separate *Agrobacterium tumefaciens* strain GV3101 cells (Weidi Biotechnology, Shanghai, China) for the infiltration of *N. benthamiana* leaves. LUC and REN activities were measured using a Dual Luciferase Reporter Assay Kit (Vazyme) and a GloMax^®^ 96 Microplate Luminometer (Promega, Madison, WI, USA). Leaves were infiltrated and the LUC: REN ratio was determined as previously described [27].

### Statistical analysis

Data were calculated and compiled using Excel 2010 (Microsoft 2010, Redmond, WA, USA). Student’s *t*-test and an analysis of variance were conducted using SPSS Statistics 26 (IBM Corp., Armonk, NY, USA). Data were recorded as the mean ± standard deviation.

## Conclusion

A wheat *Q* gene mutant carrying the *Q*^*c1*^*-Q1* allele was isolated following chemical mutagenesis. Notably, *Q*^*c1*^*-Q1* increased wheat yield through its positive effects on TKW and grain number per spike. These positive effects were not associated with a decrease in grain quality, indicating that *Q*^*c1*^*-Q1* may be useful for wheat breeding. In addition, a specific KASP marker was developed to accelerate the use of *Q*^*c1*^*-Q1* in wheat breeding programs.

## Supplementary Information

Supplementary Material 1: Figure. S1 Alignment of the genomic DNA sequences of the domesticated Q allele (Genebank No. KX580301), overexpressed Qc1 allele (KX580302), and Qc1-Q1 allele (PX626453). The microRNA172-binding site, 10 exons, and nine introns are annotated. Red arrows indicate point mutations.

Supplementary Material 2: Figure. S2 Alignment of the cDNA sequences of the domesticated Q allele, overexpressed Qc1 allele, and two transcripts (Qc1-Q1a and Qc1-Q1b) of Qc1-Q1. The microRNA172-binding site and sequences of seven conserved domains (motif 1, motif 2, nuclear localization signal, AP2 domain R1, AP2 domain R2, motif 3, and AASSGF box) are annotated. Red arrows indicate point mutations. The second intron retained in Qc1-Q1b is boxed in red.

Supplementary Material 3: Figure. S3 Alignment of the deduced amino acid sequences of the domesticated Q allele, overexpressed Qc1 allele, and two transcripts (Qc1-Q1a and Qc1-Q1b) of Qc1-Q1. Seven conserved domains (motif 1, motif 2, nuclear localization signal, AP2 domain R1, AP2 domain R2, motif 3, and AASSGF box) are indicated under the corresponding sequences. Asterisks indicate the stop codon. The red arrow indicates the amino acid change due to C3129T.

Supplementary Material 4: Figure. S4 Morphology of Q gene mutant plants (a) and schematic diagram of the generation of Q gene mutants with the genetic background of common wheat cultivar “Shumai 482” (b).

Supplementary Material 5: Figure. S5 Comparison of the intact loaves and biscuits produced using grains of SM482-Q and SM482-Qc1-Q1. (a) Loaf shape. (b) Biscuit shape. (c) Biscuit thickness.

Supplementary Material 6: Figure. S6 Q gene expression level in grains of SM482-Q (a) and SM482-Qc1-Q1 (c) at 20 DPA as determined by reverse transcription PCR. The UniGene Ta.14126 (Genebank No. BE429982) are used as the reference (c and d).

Supplementary Material 7: Figure. S7 Transient transcriptional assay of Q, QC1-Q1a, and QC1-Q1b using N. benthamiana leaves. (a) Reporters and effectors used in the assay. GAL4-BD: GAL4 DNA-binding domain; LUC: firefly luciferase; REN: Renilla luciferase. (b) Luciferase activities in effector-expressing samples. The LUC-to-REN activity (LUC:REN) ratio was used to estimate transcriptional activation by effectors. Control and VP16 were used as negative and positive controls, respectively. Data are presented as the mean ± standard deviation (n = 5).

Supplementary Material 8: Figure. S8 Comparison of plant architecture (a), grain width (d), spike morphology (c), and grain length (d) between the parents (AS1 and “Chuanmai620”) and three superior plant lines (CR1, CR2, and CR3) at GS87. CM620: “Chuanmai620”.

Supplementary Material 9: Table S1 Primers used in this study.Table S2 Comparison of the agronomic traits between the parents and superior lines under individual conditions.

Supplementary Material 10.

## Data Availability

All data generated or analysed during this study are included in this published article and its supplementary information files.

## Abbreviations

AP2: Apetala2
DPA: Days post anthesis
EMS: Ethyl methanesulfonate
GFP: Green fluorescent protein
GPC: Grain protein content
KASP: Kompetitive allele-specific PCR
RT-PCR: Reverse transcription PCR
TKW: Thousand kernel weight
UAS: Upstream activation sequence
