# Supplementary figures and images for: Q allele overexpression and alternative splicing can improve wheat yield by increasing thousand-kernel weight and grain number per spike
Source: BMC Plant Biol. 2026 Jan 24;26:331. doi: 10.1186/s12870-026-08188-4 (PMC12910859; doi:10.1186/s12870-026-08188-4)

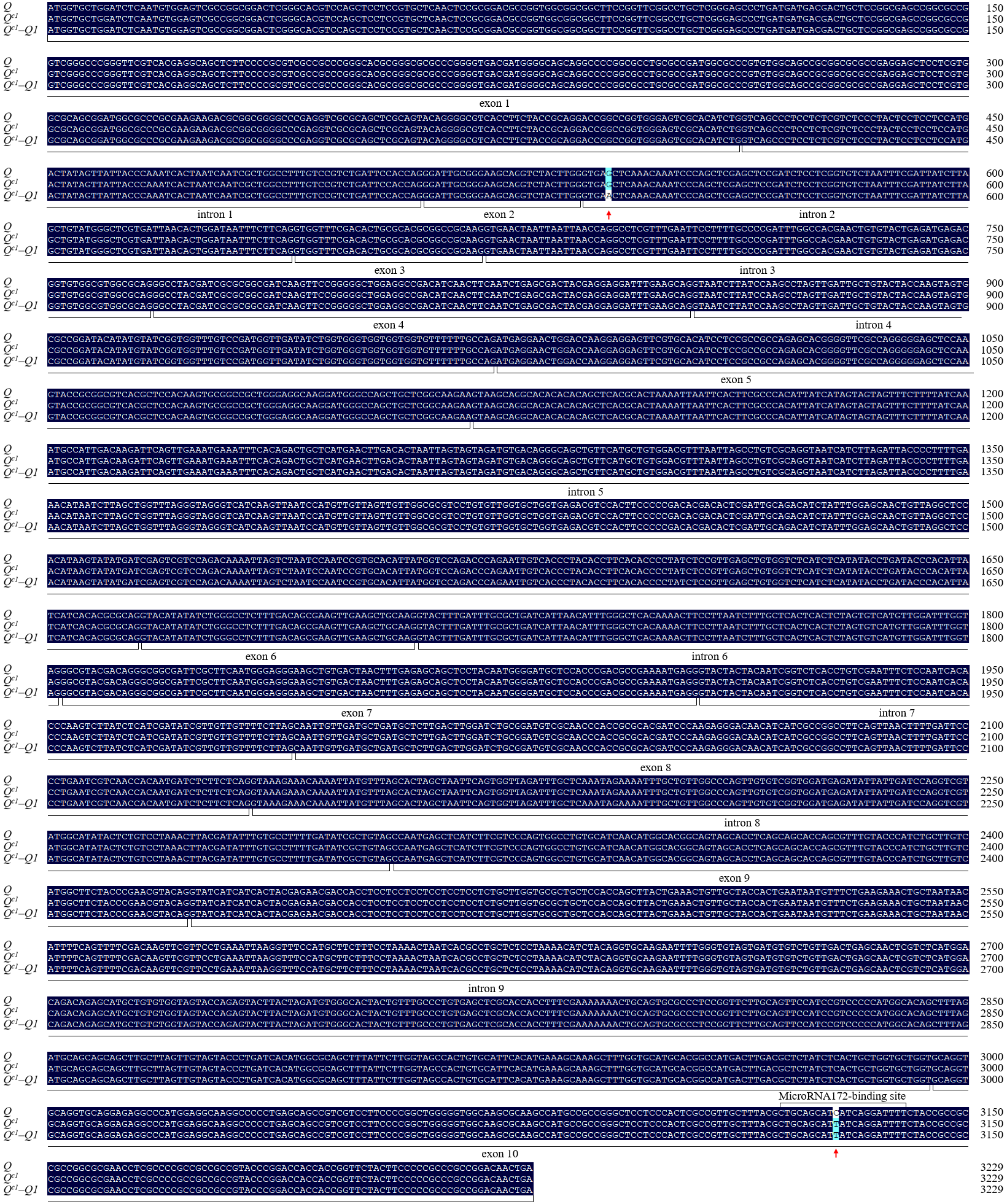

Supplement: Supplementary file 1 — Supplementary Material 1: Figure. S1 Alignment of the genomic DNA sequences of the domesticated Q allele (Genebank No. KX580301), overexpressed Qc1 allele (KX580302), and Qc1-Q1 allele (PX626453). The microRNA172-binding site, 10 exons, and nine introns are annotated. Red arrows indicate point mutations. [file 12870_2026_8188_MOESM1_ESM.tif]

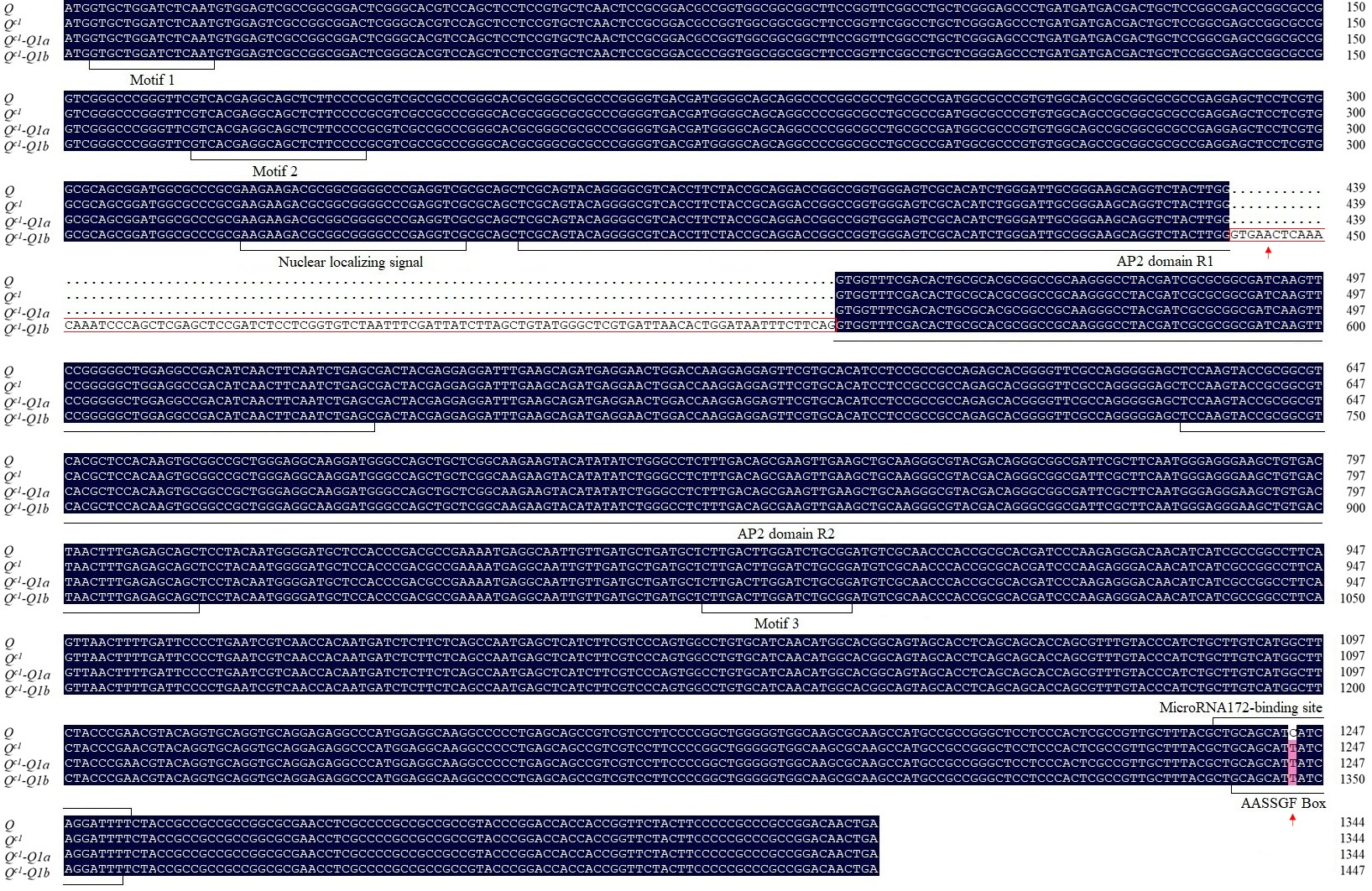

Supplement: Supplementary file 2 — Supplementary Material 2: Figure. S2 Alignment of the cDNA sequences of the domesticated Q allele, overexpressed Qc1 allele, and two transcripts (Qc1-Q1a and Qc1-Q1b) of Qc1-Q1. The microRNA172-binding site and sequences of seven conserved domains (motif 1, motif 2, nuclear localization signal, AP2 domain R1, AP2 domain R2, motif 3, and AASSGF box) are annotated. Red arrows indicate point mutations. The second intron retained in Qc1-Q1b is boxed in red. [file 12870_2026_8188_MOESM2_ESM.tif]

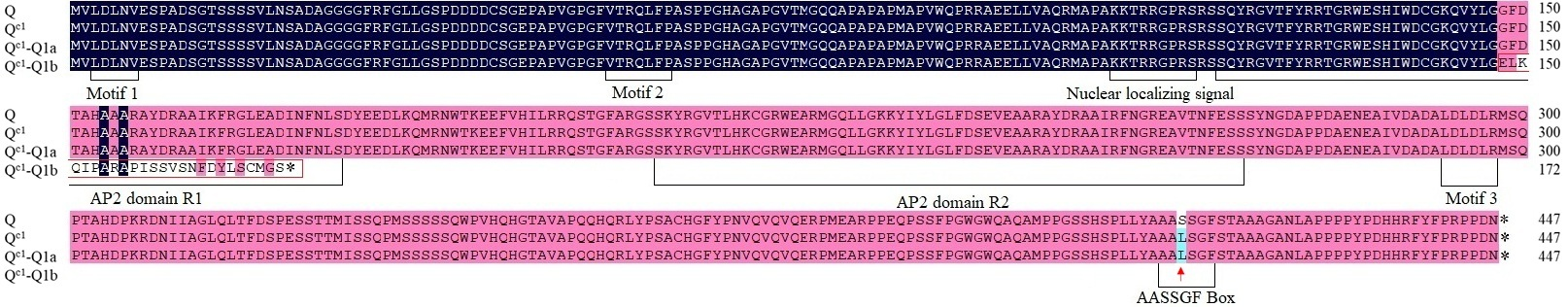

Supplement: Supplementary file 3 — Supplementary Material 3: Figure. S3 Alignment of the deduced amino acid sequences of the domesticated Q allele, overexpressed Qc1 allele, and two transcripts (Qc1-Q1a and Qc1-Q1b) of Qc1-Q1. Seven conserved domains (motif 1, motif 2, nuclear localization signal, AP2 domain R1, AP2 domain R2, motif 3, and AASSGF box) are indicated under the corresponding sequences. Asterisks indicate the stop codon. The red arrow indicates the amino acid change due to C3129T. [file 12870_2026_8188_MOESM3_ESM.tif]

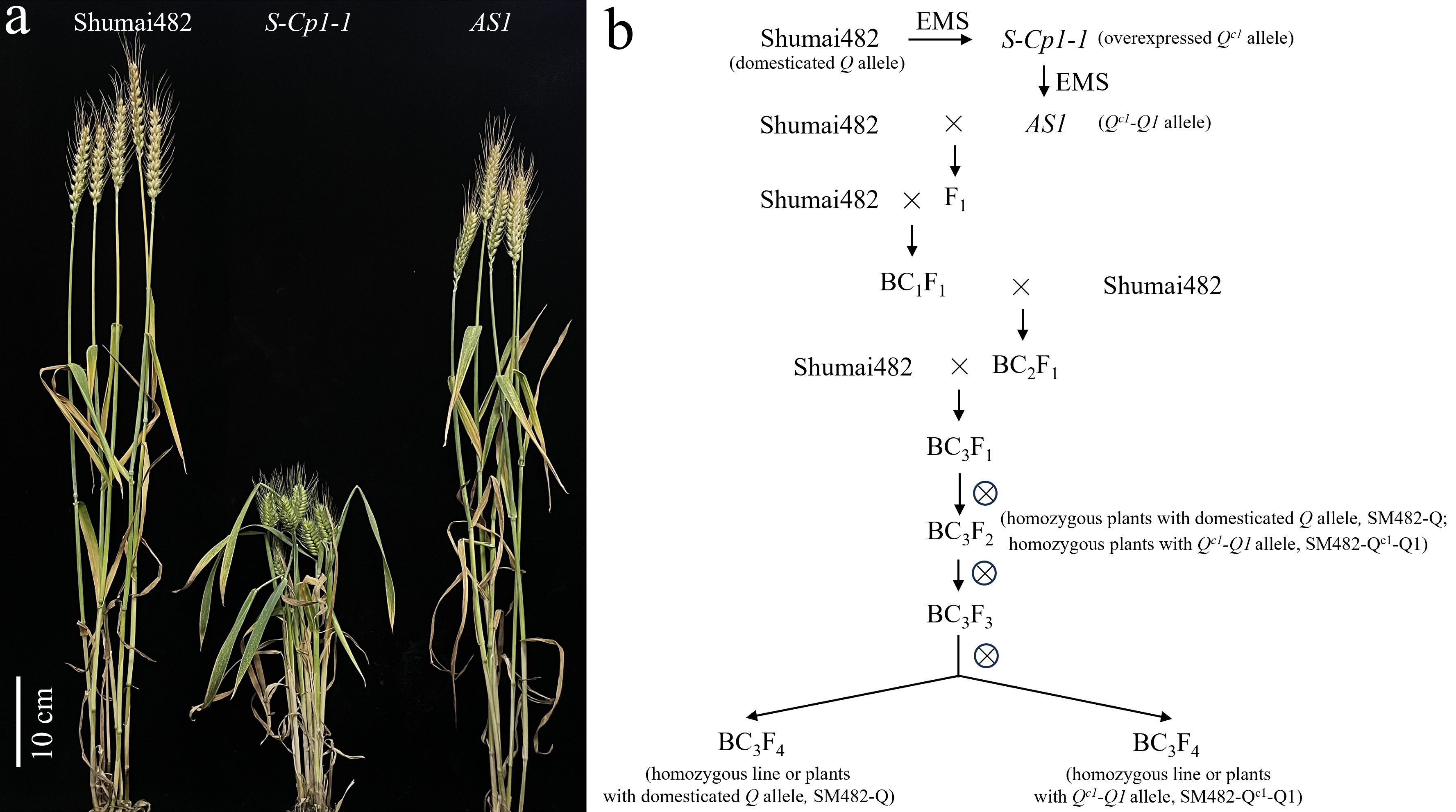

Supplement: Supplementary file 4 — Supplementary Material 4: Figure. S4 Morphology of Q gene mutant plants (a) and schematic diagram of the generation of Q gene mutants with the genetic background of common wheat cultivar “Shumai 482” (b). [file 12870_2026_8188_MOESM4_ESM.tif]

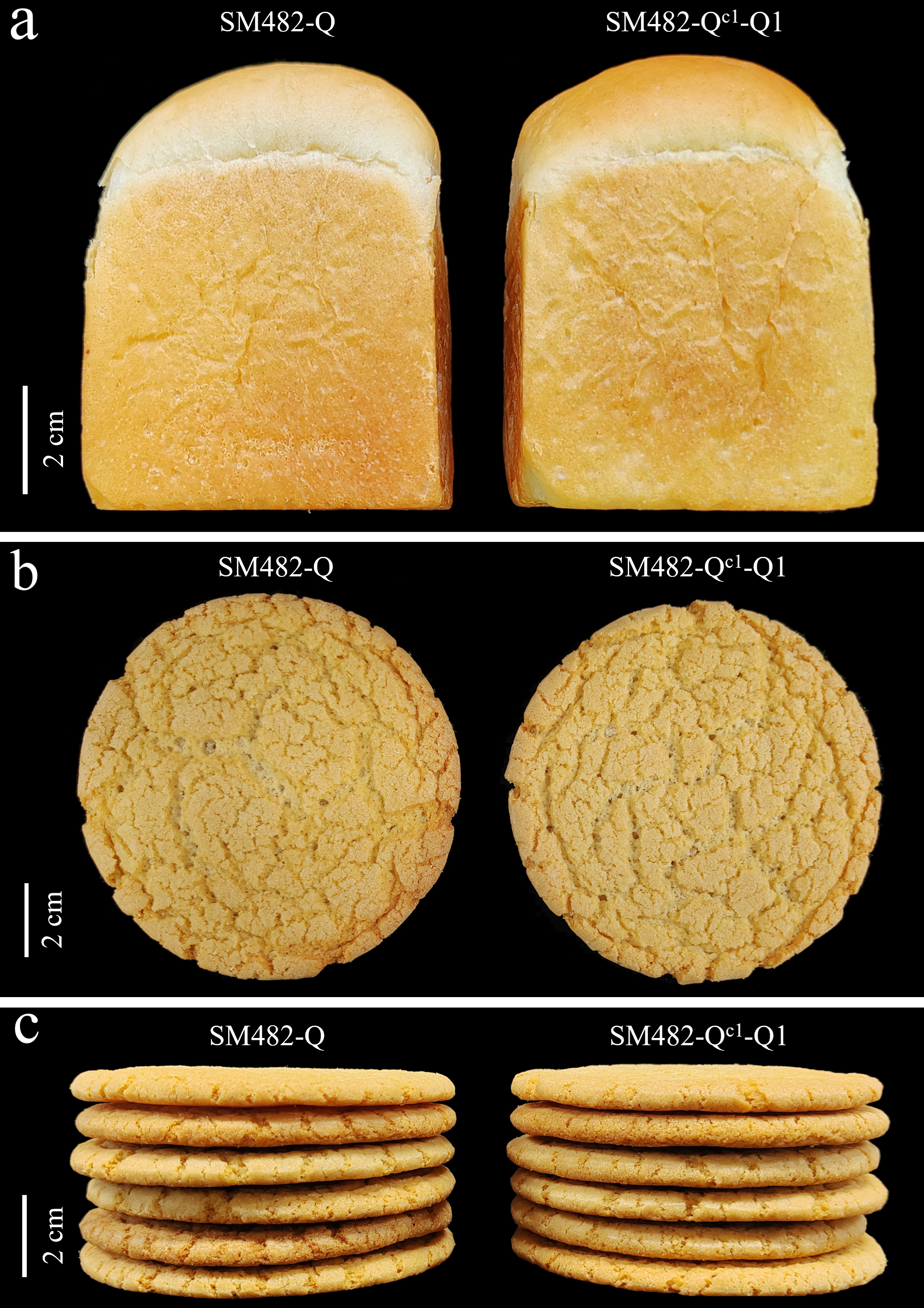

Supplement: Supplementary file 5 — Supplementary Material 5: Figure. S5 Comparison of the intact loaves and biscuits produced using grains of SM482-Q and SM482-Qc1-Q1. (a) Loaf shape. (b) Biscuit shape. (c) Biscuit thickness. [file 12870_2026_8188_MOESM5_ESM.tif]

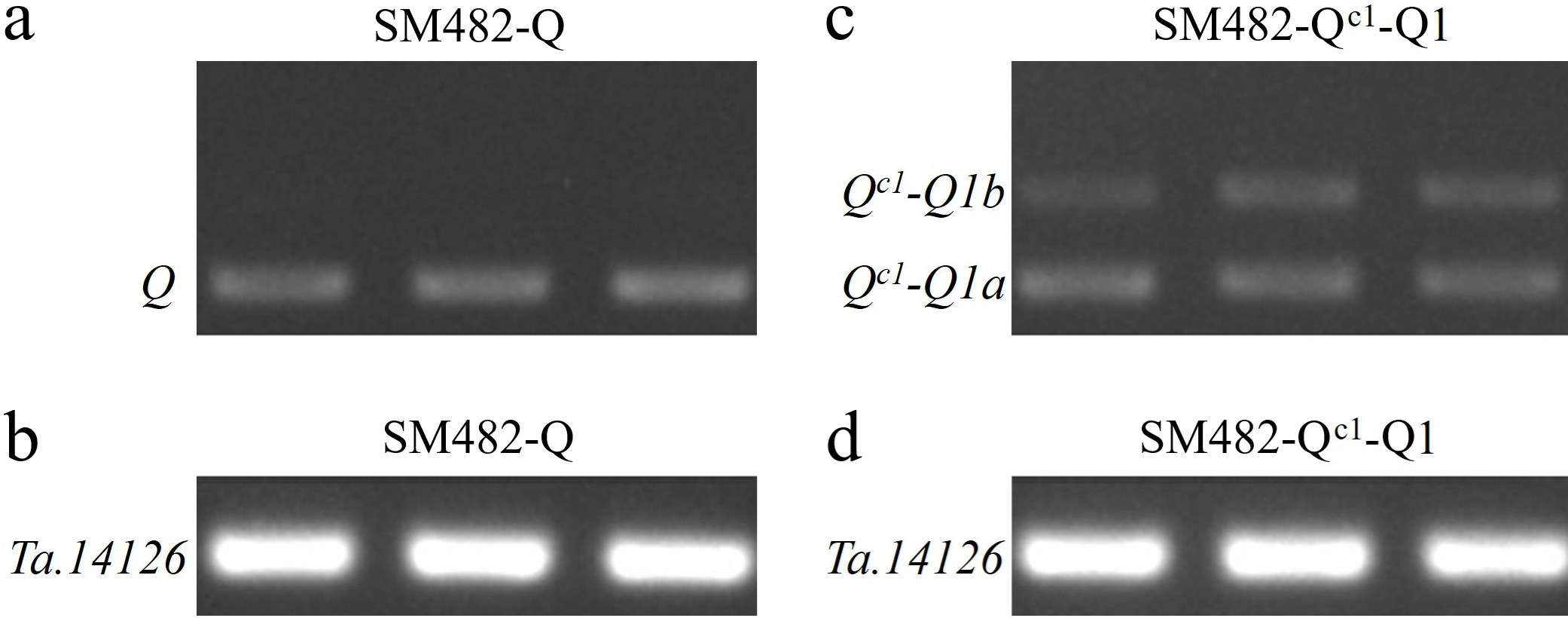

Supplement: Supplementary file 6 — Supplementary Material 6: Figure. S6 Q gene expression level in grains of SM482-Q (a) and SM482-Qc1-Q1 (c) at 20 DPA as determined by reverse transcription PCR. The UniGene Ta.14126 (Genebank No. BE429982) are used as the reference (c and d). [file 12870_2026_8188_MOESM6_ESM.tif]

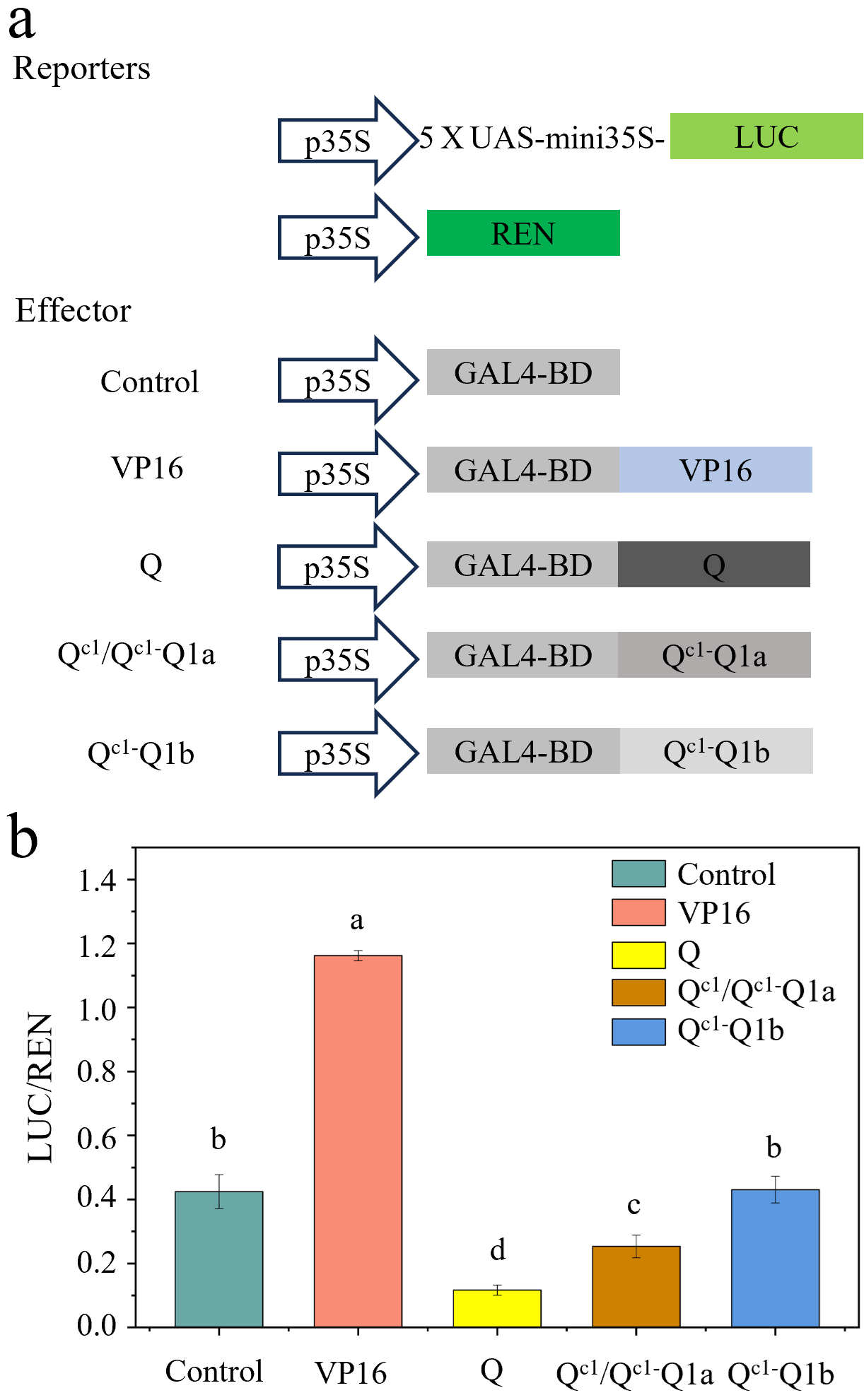

Supplement: Supplementary file 7 — Supplementary Material 7: Figure. S7 Transient transcriptional assay of Q, QC1-Q1a, and QC1-Q1b using N. benthamiana leaves. (a) Reporters and effectors used in the assay. GAL4-BD: GAL4 DNA-binding domain; LUC: firefly luciferase; REN: Renilla luciferase. (b) Luciferase activities in effector-expressing samples. The LUC-to-REN activity (LUC:REN) ratio was used to estimate transcriptional activation by effectors. Control and VP16 were used as negative and positive controls, respectively. Data are presented as the mean ± standard deviation (n = 5). [file 12870_2026_8188_MOESM7_ESM.tif]

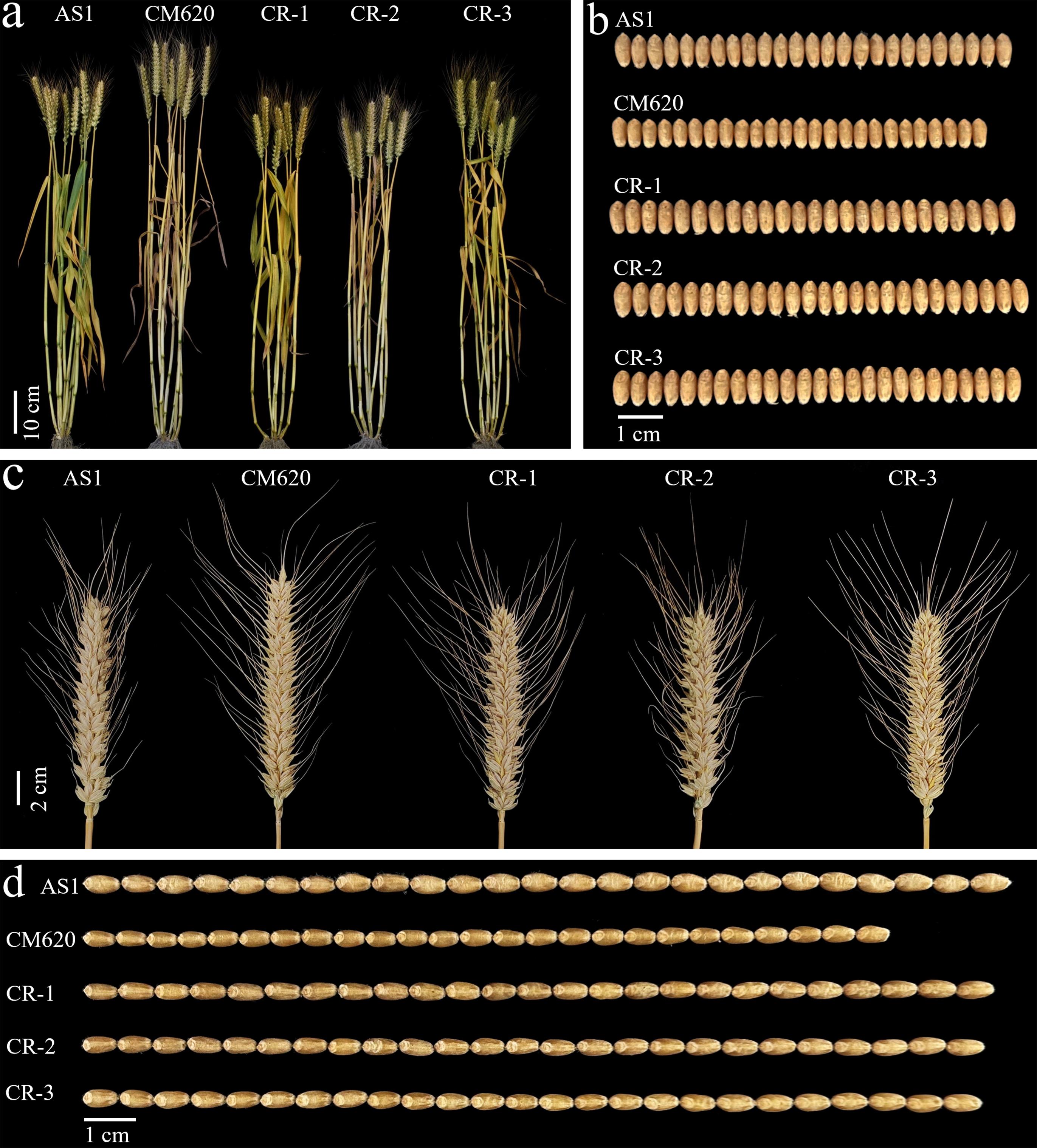

Supplement: Supplementary file 8 — Supplementary Material 8: Figure. S8 Comparison of plant architecture (a), grain width (d), spike morphology (c), and grain length (d) between the parents (AS1 and “Chuanmai620”) and three superior plant lines (CR1, CR2, and CR3) at GS87. CM620: “Chuanmai620”. [file 12870_2026_8188_MOESM8_ESM.tif]

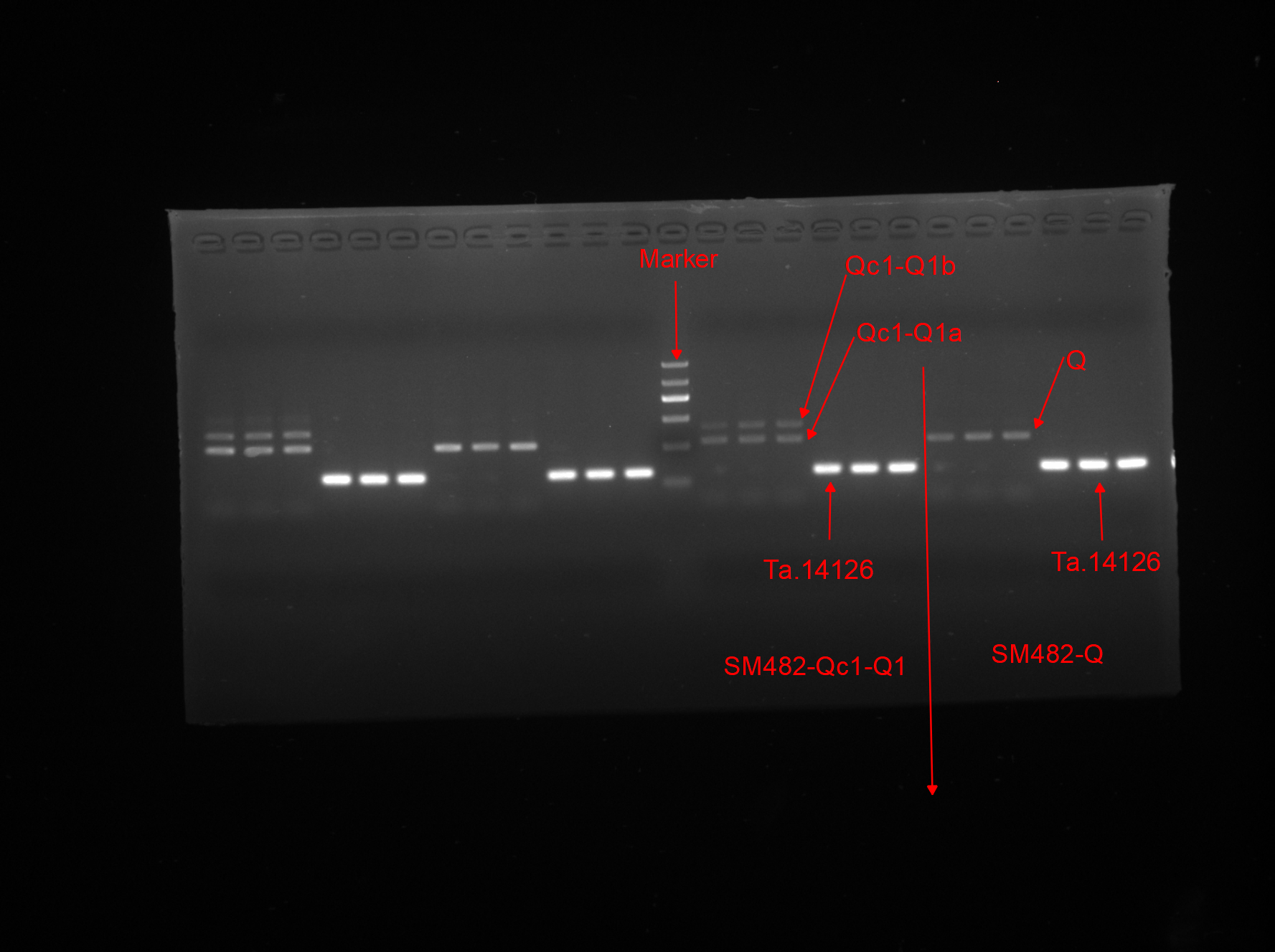

Supplement: Supplementary file 10 — Supplementary Material 10. [file 12870_2026_8188_MOESM10_ESM.tif]
